# Fidelity of HIV programme implementation by community health workers in rural Mopani district, South Africa: a community survey

**DOI:** 10.1186/s12889-018-5927-2

**Published:** 2018-09-06

**Authors:** Nireshni Naidoo, Jean P. Railton, Sellina N. Khosa, Nthabiseng Matlakala, Gert Marincowitz, James A. McIntyre, Helen E. Struthers, Jude Igumbor, Remco P. H. Peters

**Affiliations:** 1 Anova Health Institute, PostNet Suite 242, Private Bag X30500, 2041 Houghton, Johannesburg, South Africa; 2 Division of Epidemiology and Biostatistics, School of Public Health, Faculty of Health Sciences, University of the Witwatersrand, Johannesburg, South Africa; 3 Mopani District Specialist Team, Department of Health, Giyani, Limpopo Province South Africa; 4 School of Public Health & Family Medicine, University of Cape Town, Cape Town, South Africa; 5 Division of Infectious Diseases & HIV Medicine, Department of Medicine, University of Cape Town, Cape Town, South Africa; 6 Department of Medical Microbiology, School of Public Health and Primary Care (CAPHRI), Maastricht University Medical Centre (MUMC+), Maastricht, The Netherlands

**Keywords:** Community health workers, HIV, Implementation, Community, Public health

## Abstract

**Background:**

South Africa has implemented a community health programme delivered by community health workers (CHWs) to strengthen primary healthcare services. Provision of community Human Immunodeficiency Virus (HIV) services constitutes an important component of this programme. To support effectiveness, we assessed fidelity of HIV programme implementation by CHWs from the community’s perspective in a rural South African setting.

**Methods:**

A cross-sectional study was conducted targeting 900 randomly selected households in twelve wards of two sub-districts (Greater Giyani and Greater Letaba) of Mopani District (Limpopo Province, South Africa). Questionnaires were administered to the traditionally most appropriate adult member of the household. Included were questions related to the four standard components to measure implementation fidelity against local guidelines: coverage, frequency, duration and content of HIV programme implementation.

**Results:**

Participants were enrolled at 534 households; in most other cases there was nobody or no adult member at home (*n* = 291). Reported coverage of 55% (141/253) and a frequency of 47% (66/140) were higher in Greater Giyani as compared to Greater Letaba (44%; 122/278 and 29%; 33/112, respectively, *p* = 0.007 for both comparisons). Coverage was not associated with the distance from the participant’s household to the facility (*p* = 0.93). Duration of programme delivery was reported to be high, where all CHW visits (253/253; 100%) were conducted within the last 6 months and the content delivered was adequate (242/253; 96%). Individuals reporting a CHW visit were more likely to know their HIV status than those not visited (OR = 2.0; 95% CI 1.06–3.8; *p* = 0.032). Among those visited by the CHW discussion of HIV was associated with knowing the HIV status (OR = 2.2; 95% CI 1.02–4.6; *p* = 0.044); in particular for women (OR = 2.9; 95% CI 1.5–5.4; *p* = 0.001).

**Conclusions:**

This study demonstrates promising HIV programme implementation fidelity by CHWs in rural South Africa. Programme coverage and frequency should be improved whilst maintaining the good levels of duration and content. Resource investment, strengthening of operational structure, and research to identify other facilitators of programme implementation are warranted to improve programme effectiveness and impact.

## Background

Community health programmes have strong potential to strengthen primary health services in low- and middle income countries (LMICs) [1]. The value of these programmes has been demonstrated for maternal, child, and mental healthcare in various settings in LMICs. For example, studies from South Africa show that CHWs have been successful in improving maternal and child health outcomes [2], and providing a social support system [3]. Reviews of quantitative and qualitative studies show that CHWs can provide an important human resource capacity and have the potential to contribute to HIV services in sub-Saharan Africa [4, 5]. There is growing evidence on successful scale up and integration of community-based programmes into national health systems [6–8]. However, despite its strong potential, only limited data are available with regard to provision of HIV services at community level [9, 10].

South Africa, the country with the largest HIV programme in the world, has implemented a community health programme since 2012 with the aim to improve access to and provision of high-quality primary healthcare (PHC) services [11]. South Africa’s community health programme is delivered through ward-based outreach teams (WBOTs), each comprising a professional nurse that serves as team leader and up to five community health workers (CHWs) [11]. The local guidelines stipulate that the team of five CHWs should support a population of 7660 individuals in total [11]. The services provided by the CHWs include general health education, health status monitoring, and referral of individuals in need of care to the PHC facility [11].

There is a strong programmatic focus on vulnerable populations, including individuals with chronic illnesses; HIV or tuberculosis (TB); pregnant women; as well as screening for malnutrition and gastroenteritis; and checking immunisation, vitamin A and deworming status in children.

Provision of HIV services constitutes an important component in the community health programme. The CHWs contribute to HIV programme delivery through providing health education to prevent HIV infection, identifying individuals that need to test for HIV, referring HIV-infected individuals not yet in care to start antiretroviral therapy (ART), providing adherence support to those on ART, tracing and referral of HIV-infected individuals that have been lost to the ART programme, and identifying individuals who have clinically failed on ART and require further assessment [11]. Visits to HIV-infected individuals should happen on a monthly basis while assessment of HIV risk and potential referral for testing should happen at least annually [11].

Regular assessment and monitoring of implementation performance of any health programme is essential to obtain maximum impact of its implementation and to ensure that the programme meets its intended goals [12]. The same holds true for the HIV programme as provided by CHWs. However, to the best of our knowledge there is no record of a comprehensive assessment of implementation of this programme in South Africa [13]. Studies from other countries, not addressing HIV, show that lack of such information could negatively affect programme effectiveness [2–4]. Knowledge of how and why a community health programme works, or does not work, in terms of implementation is required to optimise programme implementation before successful scale-up [1, 6, 14].

An important method to assess implementation of health programmes is to measure fidelity to the intended programme specifics. Fidelity to programme implementation is generally measured on four aspects: ‘coverage’ (services provided to everyone that is supposed to receive these services), ‘frequency’ (services provided by the prescribed frequency), ‘duration’ (service delivery that is uninterrupted) and ‘content’ (the correct services provided) [15]. In our context, this means measurement of HIV programme implementation by CHWs of these four components against the programme specification in the local government guidelines [11]. In this study we aim to measure fidelity to HIV programme implementation as provided by CHWs from the community’s perspective in rural Mopani District, South Africa. We conducted a community survey to obtain insight into the current status of programme implementation and to identify areas that warrant strengthening to improve effectiveness of HIV service delivery by CHWs.

## Methods

### Study setting and design

The community health programme was initiated in Mopani district in 2013 and, as of mid-2016, is provided by 149 WBOTs that cover 123 of the 125 wards (98%) and 75% of registered households. [16]. All CHWs are female. A cross-sectional study was conducted from May to July 2016 in two sub-districts (Greater Giyani and Greater Letaba) of the Mopani district, Limpopo province, South Africa. Mopani district is one of the most rural districts in the country and is considered to be one of the main infrastructure intervention areas for economic transformation in South Africa [17]. There are 83,225 and 54,228 households in Greater Giyani and Greater Letaba sub-district supported by 37 and 24 WBOTs, respectively. The WBOTs in these sub-districts have operational differences such as the number of CHWs deployed, workload, and team performance.

We purposively selected the communities living in twelve wards, six per sub-district. These wards were selected as each was the single ward draining to a PHC facility (some facilities have multiple wards draining into them) and the catchment population was of similar size (+/− 45,000 individuals). Each of the twelve wards is supported by a single WBOT that has divided the ward into sections. Households in each section are supported by an individual CHW. For this study we randomly selected three of the sections in each ward (36 in total). We then randomly selected 25 households in each section by manually pinning households in Google™ Earth Pro. The Global Positioning System (GPS) coordinates of each selected household was recorded and given to the study team. We used coverage, i.e. report of a CHW household visit by the participant, as the main measure of outcome. Based on an estimated coverage of 80%, using a confidence level of 95%, and power of 80%, we calculated a sample size of 734 individuals as adequate. Assuming a response rate of 80%, a sample size of 900 was used. Ethical approval was obtained from the Human Research Ethics Committee at the University of Witwatersrand, Johannesburg, South Africa (Reference number: M1611111) as well as the Limpopo Provincial Health Research Committee.

### Study procedures

The study team visited selected households using maps and GPS tracker to find the correct location. Individuals found at home were approached to participate and screened for eligibility. Individuals had to be adult (> 18 years) to participate; in the case that there were multiple people at home, the most senior individual, as culturally appropriate, was interviewed. Following written informed consent, a questionnaire was administered that included questions about the individual’s demographic details, interactions with and perceptions of their CHW as well as the services received from them.

### Measurement of implementation fidelity

Implementation fidelity was assessed by measuring content, coverage, frequency, and duration in relation to the provincial guidelines that are currently in place [11]. *Coverage*, ‘reach’, was measured by the proportion of households that reported CHW visits ever. *Frequency* was assessed by determining the proportion of households that received CHW visits according to the required schedule (at least once a month in case of vulnerable household). *Duration* of implementation refers to the need for ongoing service delivery, i.e. no major programme interruptions; we defined duration as high in case of CHW visit < 6 months ago or low for CHW visit > 6 months ago. Finally, *content* was measured by the proportion of individuals reporting HIV services delivered by CHWs that were deemed by the researchers in line with the guidelines, including HIV health education and referrals [11].

### Statistical analysis

Data were collected using structured interview guides and double-captured into a database (EpiInfo™). Validity checks were done to ensure completeness and to identify consistency and range errors. Socio-demographic characteristics of the households are described using proportions with confidence intervals and means with standard deviation. We determined factors associated with a CHW visit using univariate logistic regression. Variables with a *p* value ≤0.1 on univariate logistic regression were included in the multivariate logistic regression model as well as sub-district as expected confounding variable. We included social grant in our analysis as provided by the South African government to various groups (pensioners, disabled individuals, and child support).

## Results

### Description of the study population

**Fig. 1:**
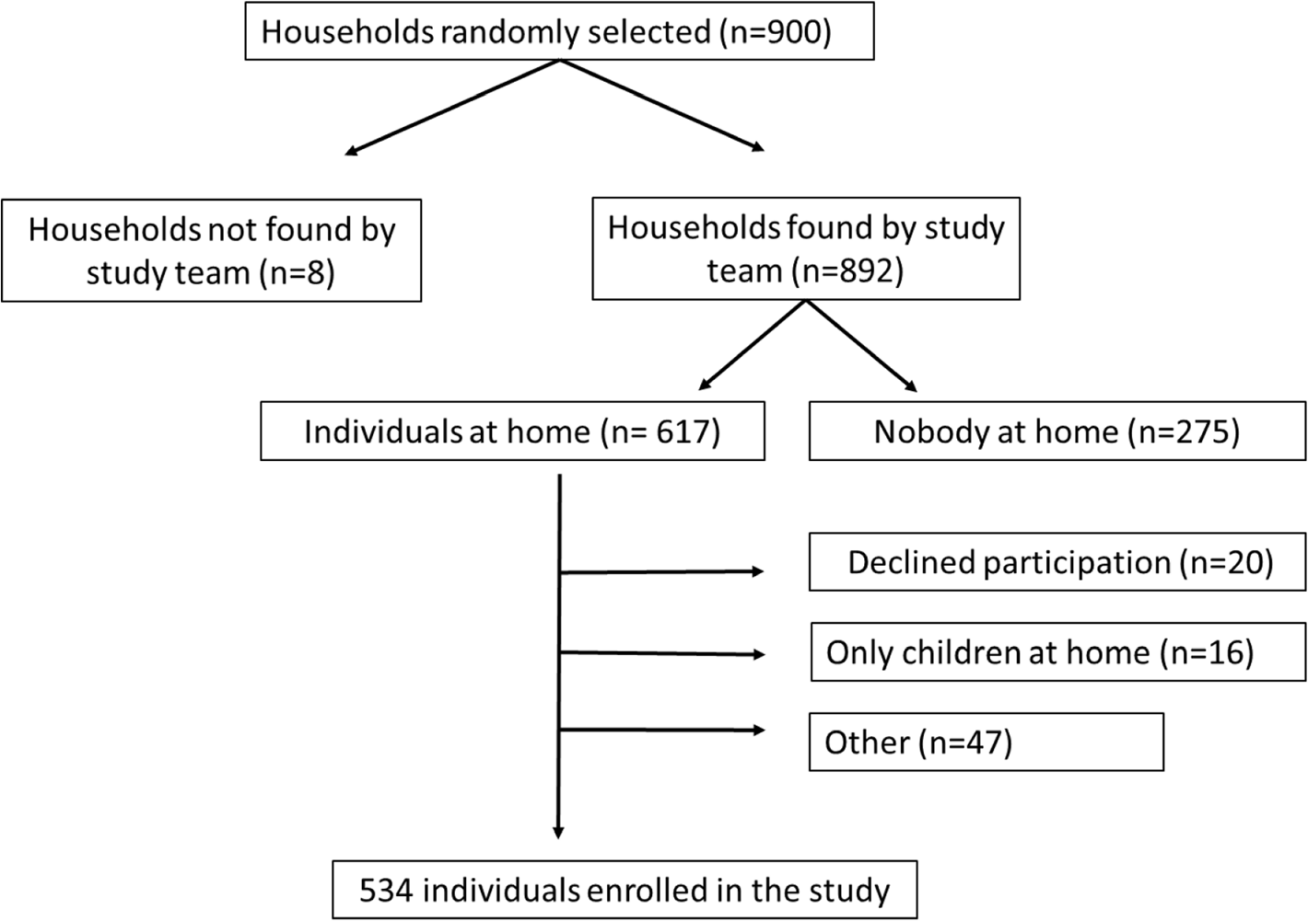
Participant enrolment in the study

The study team managed to visit 99% of the households (892/900) randomly selected for this study; in the other 8 cases there was no longer a house at the location identified on the map. Participants were enrolled at 534/892 (60%) households; there was no difference in enrolment between Greater Giyani (49.6%) and Greater Letaba (50.3%) sub-districts (*p* = 0.79). At the non-included households (358), there was nobody at home in 275 (31%) cases or only a child at home (16; 1.8%). Participation was declined at 47 households (5.3%) for other reasons (Fig. 1). The majority of participants was female (410/534; 77%) and median age was 46 years (range 18–96) (Table 1). Social grants were the main source of income reported by participants in both sub-districts, 52% (137/265) and 43% (117/269) in Greater Giyani and Greater Letaba respectively (*p* = 0.028).

**Table 1:** Characteristics of the study population (*n* = 534)

Variable		Greater Giyani (*N* = 265)	Greater Letaba (*N* = 269)	Total
Gender	Male	61 (23%)	63 (23%)	124
	Female	204 (77%)	206 (77%)	410
Source of income	Formal employment	63 (24%)	92 (34%)	155
	Informal work	56 (21%)	58 (22%)	114
	Social grants	137 (52%)	117 (43%)	254
	Other	9 (3%)	2 (1%)	11
Primary member in the household that takes of the children	Mother	77 (29%)	79 (29%)	156
	Father	76 (29%)	74 (28%)	150
	Grandmother	50 (19%)	60 (22%)	110
	Other	62 (23%)	56 (21%)	118
Age of participants (years)	18–34	88 (33%)	90 (34%)	178
	35–59	90 (34%)	119 (44%)	209
	≥60	87 (33%)	60 (22%)	147
^a^Median number of individuals living in household		5 (0–10)	6 (0–10)	5.5
^a^Median distance from participant’s house to the clinic (km)		1.4 (0–13)	1.6 (0–11)	1.5

^a^Numbers presented in median (range)

### Implementation fidelity: measurement of coverage, frequency, and duration

In terms of coverage, participants from 253/534 (47%) households reported to have ever been visited by a CHW (Table 2), of which 100% (253/253) were visited in the last 6 months. A CHW visit was more often reported in Greater Giyani (55%) than in Greater Letaba (45%) sub-district (OR = 1.6; 95% CI 1.14–2.27; *p* = 0.007). This is possibly due to the fact that there are 54 and 36 CHWs deployed on Greater Giyani and Greater Letaba, respectively, and that Greater Giyani has better organisational structures. Individuals 60 years and older were more likely to be visited by a CHW as compared to individuals 18 to 34 years of age (OR = 1.7; 95% CI 1.08–2.77; *p* = 0.022. There was no association of reporting a CHW visit with distance between household and nearest facility (1.4 km for visited vs. 1.6 km for not visited; *p* = 0.93). We plotted households on the map by status of CHW visit reported, and did not observe any obvious patterns or clusters that could indicate other geographic factors. Most participants who reported a CHW visit were happy with such visit (192/253; 76%) whereas a large proportion of participants not visited by a CHW would have liked such a visit (206/278; 74%). Participants who had been visited by a CHW were more likely to know their HIV status (238/253; 94%) compared to those not visited (245/278; 88%) (OR = 2.0; 95% CI 1.06–3.8; *p* = 0.032); there was no statistically significant difference between whether or not a household was visited and gender (OR = 1.4; 95% CI 0.92–2.08; *p* = 0.118).

**Table 2:** Univariate and multivariate analysis of factors associated with participant reporting CHW visit

Variable		Visited by CHW	Not visited by CHW	Unadjusted OR (95% CI)	*P*-value	Adjusted OR (95% CI)	*P-*value
Number		253 (48%)	278 (52%)				
Sub-district	Giyani	141 (55%)	122 (44%)	**1.7 (1.14–2.27)**	**0.007**	**1.6 (1.14–2.27)**	**0.007**
	Letaba	112 (45%)	156 (56%)	Ref	Ref	Ref	Ref
Distance to clinic (km)		1.4 (0–13)	1.6 (0–11)		0.934	–	–
Gender	Male	51 (20%)	72 (26%)	Ref	Ref	–-	–
	Female	202 (80%)	206 (74%)	1.4 (0.92–2.08)	0.118	–	–
Age-group (years)	18–34 years	75 (30%)	100 (36%)	Ref	Ref	Ref	Ref
	35–59 years	95 (37%)	114 (41%)	1.1 (0.74–1.67)	0.610	1.2 (0.76–1.76)	0.503
	≥60 years	83 (33%)	64 (23%)	**1.7 (1.11–2.69)**	**0.015**	**1.7 (1.08–2.77)**	**0.022**
Source of income	Formal	70 (28%)	83 (30%)	Ref	Ref	–	–
	Informal	49 (19%)	65 (23%)	0.9 (0.52–1.41)	0.546	–	–
	Grants	129 (51%)	124 (45%)	1.2 (0.82–1.87)	0.313	–	–
Participant knowledge of HIV	Yes	229 (91%)	231 (83%)	**2.1 (1.08–3.90)**	**0.029**	**2.0 (1.06–3.8)**	**0.032**
	No	15 (6%)	31 (11%)	Ref	Ref	Ref	Ref -
	Unknown	9 (4%)	15 (5%)	–	–	–	–

*Abbreviations*: *CHW* community health workers, *OR* odds ratio, *CI* confidence interval

^*^Participants that refused to answer whether they had been visited by a CHW or not were omitted from the analysis (*n* = 3). Statistically significant factors in bold

In terms of frequency, 39% (99/253) of participants overall reported that the CHW visit had occurred in the previous month as per provincial guideline. However, frequency of a CHW visit in the previous month was 47% (66/140) in Greater Giyani and only 29% (33/112) in Greater Letaba district (*p* = 0.007). There was no association of any demographic factors with frequency. In terms of duration of HIV programme implementation, all participants who had been visited by a CHW (253/253; 100%) reported high duration in both sub-districts (last visit < 6 months ago).

### Implementation fidelity: measurement of content

Ninety-six percent (242/253) of participants visited by a CHW had discussed any type of health issue during the last visit: 67% (170/253) reported having discussed HIV with the CHW, 66% (167/253) TB, 74% (187/253) chronic illnesses other than HIV, 11% (28/253) pregnancy, and 55% (139/253) other health-related issues. Most participants, 89% (225/253), were satisfied with the content of services rendered by the CHW.

Participants were twice as likely to know their HIV status if HIV had been discussed with them by the CHW compared to those with whom HIV was not discussed (OR = 2.2; 95% CI 1.02–4.60; *p* = 0.044). Females were twice as likely to know their HIV status as compared to men (OR = 2.9; 95% CI 1.5–5.4; *p* = 0.001). There was no association for women of knowing their HIV status and having discussed HIV with CHWs (OR = 1.5; 95% CI 0.6–3.5; *p* = 0.401); for men there was a positive trend (OR = 4.2; 95% CI 0.9–19.1; *p* = 0.066). In terms of referring participants for further screening or treatment, 20% (51/253) of participants reported that a CHW had referred the participant or another household member to the nearest facility during the last visit for HIV testing (23/51; 45%), TB screening (7/51; 14%) or an assessment of other chronic illnesses (20/51; 39%) and pregnancy testing (1/51; 1%). Of these referrals, 88% (45/51) reported to have visited the clinic for healthcare.

## Discussion

This is the first study to measure fidelity of implementation of the HIV programme as provided by CHWs in rural South Africa. We are not aware of any other HIV programme with fidelity data from similar settings to provide a comparison to our observations. However, when compared to other South African and global studies measuring CHW chronic disease services other than HIV, we observed similar findings with regard to duration and content, and similarly found that coverage and frequency should be improved [18–20].

In terms of coverage and frequency, we measured a 47% coverage of households from which the participant reported a CHW visit at any point in time, despite the policy that all households ought to be visited on an annual basis to assess vulnerability status. This is lower than reported by studies of chronic diseases that showed coverage in the range of 57% to 74% [19, 20], but good compared to a systematic review of 38 studies that reported 18% coverage for community health programmes overall [18]. In addition, frequency of visits was relatively low at 39%, but this was similar to other studies [18]. Increasing coverage and frequency of CHW visits could directly impact on the HIV programme as participants who had been visited by CHWs were more likely to have discussed HIV and know their HIV status than those that had not been seen by a CHW. Various factors may have impacted on coverage and frequency in our area. First, operational factors may play a role: coverage was higher in Greater Giyani sub-district where in our opinion the programme stronger managed and where there are on average more CHWs employed in each community than in Greater Letaba (resulting in lower number of households covered by each CHW). Second, an important factor to consider is the capacity of an individual CHW to visit all allocated households in the section within the set timeframe. It is possible that in our rural area, the target number of 270 households, which in practice becomes 250–400 households, is too large to cover within the specific programme deliverables. Reduction of the number of households allocated to each CHW by increasing the number of CHWs per ward should be considered to improve coverage [11]. Third, geographic factors such as houses located in rocky ad mountainous areas may impact on accessibility of houses to CHWs. In addition, challenging terrain would likely take the CHW more time to reach a household, limiting the number of households that can be visited during a day. Although an effect cannot be ruled out, we did not observe any obvious patterns or clusters when looking at households reporting visits compared to those not visited. Distance between the household and nearest PHC facility was not associated with the report of a CHW visit as most houses were within walking distance to the facility. Despite the need to improve coverage and frequency, we observed high appreciation of CHW visits by the community; most participants were happy with the CHW visits and services provided, whereas most of those that had not been visited by the CHW would really appreciate a visit. We did not observe a difference between male and female respondents with regards to interest in and appreciation of CHW visits. This contradicts findings from another report from Pakistan that suggests that men are unwilling to interact with the female CHWs [21].

In terms of duration and content, we measured high duration (100%) of programme implementation among those visited by the CHWs as compared 39% reported the aforementioned systematic review of 38 studies [18]. This suggests that services are relatively continuous and uninterrupted once there is a structural relationship between household members and CHWs. In addition, the content of service provision for HIV was 67%) and no major gaps were identified. This is good when compared to a systematic review of 38 studies that reported content provision of chronic diseases services in the range of 40% to 66% [18, 19]. This indirectly shows the impact of the intensive CHW training programme (phase 1 and phase 2 of the national curriculum) that was conducted in Mopani District [11]. Participants with whom HIV was discussed were more likely to know their HIV status suggesting a direct link between CHW activities and HIV programme performance. We did not distinguish between HIV-infected and HIV-uninfected participants. However, HIV should be discussed with all household members.

This study shows that despite limited resources, there is a reasonably good implementation of HIV programme activities by CHWs in our rural South African district. Although coverage and frequency should be improved, there is good content and duration of service provision to those that are reached by the CHWs. This supports the high promise of CHW implementation of the HIV programme on a structural basis. Resource investment in the CHW programme, through increasing the number of CHWs and strengthening operational structures, may have to be expanded to achieve the programme’s full potential and effectiveness, the sub-district with the lower resources also scored lower in implementation fidelity. These findings are consistent with other studies showing that adequate staffing, resource provision, and a human resource management approach are imperative for successful implementation of community-based programmes [22, 23]. In light of this, the use of human resource strategies such as clarification of roles, job satisfaction, and adequate remuneration should be considered to improve the South African CBHP. Although operational determinants may play a role, other factors that contribute to the programme success or provide a barrier to successful coverage and frequency need to be determined within a local context, in order to further improve implementation fidelity. Moreover, alternate strategies should be considered to reach the large proportion of households where we found no one at home, such as worksite wellness and local media initiatives [24].

This study has several limitations. Firstly, several types of bias may have occurred including recall and desirability bias. Selection bias may have also occurred as there was nobody at home at a substantial proportion of households. Possibly, these represent higher socio-economic group as they may be at work during the day. Although we recruited more women than men, there was no difference in their response. The response rate could have possibly been increased by visiting households multiple times instead of once. However, this was not feasible for operational reasons. Secondly, we have measured implementation fidelity from the community’s (recipient) perspective. Triangulation of our findings with similar measurement from the CHW’s perspective could have strengthened our findings, but resources did not permit this. We did not allocate a health vulnerability status to the households included in this study, which would have allowed for a more precise assessment of some components of implementation fidelity. This was due to the fact that household registration data, including vulnerability status, were not available through the CHWs at the time of study and could not be reliably collected as part of the questionnaire. For operational purpose, we collected data on CHW visits within the last 6 months as opposed to the recommended 12 months, which may have resulted in an underestimation of frequency. Finally, a time component was not included in the question where individuals were asked if they knew their HIV status.

## Conclusions

In conclusion, this study shows that implementation fidelity of the HIV programme as provided by CHWs in a rural South Africa district is similar to other CHW programmes that report on chronic illnesses, other than HIV. There is room for improvement, in particular of coverage and resource investment is required to increase frequency of household visits. While this study informs policy makers on specific programme areas to improve this CBHP, further quantitative and qualitative research in similar contexts is required to further gauge barriers and facilitators to the fidelity of CHBP implementation.

## Abbreviations

ART: Antiretroviral therapy
CHWs: Community health workers
GPS: Global Positioning System
HIV: Human Immunodeficiency Virus
LMICs: Low-and middle income countries
PHC: Primary healthcare
TB: Tuberculosis
WBOTs: Ward-based outreach teams
